# Design and Application of Antimicrobial Peptide Conjugates

**DOI:** 10.3390/ijms17050701

**Published:** 2016-05-11

**Authors:** Andre Reinhardt, Ines Neundorf

**Affiliations:** Department of Chemistry, Institute of Biochemistry, University of Cologne, Zuelpicher Str. 47, D-50674 Cologne, Germany; andre.reinhardt1@gmx.de

**Keywords:** antimicrobial peptides, antimicrobial peptide conjugates, antibiotics, photosensitizer, nanoparticles, organometallic complexes

## Abstract

Antimicrobial peptides (AMPs) are an interesting class of antibiotics characterized by their unique antibiotic activity and lower propensity for developing resistance compared to common antibiotics. They belong to the class of membrane-active peptides and usually act selectively against bacteria, fungi and protozoans. AMPs, but also peptide conjugates containing AMPs, have come more and more into the focus of research during the last few years. Within this article, recent work on AMP conjugates is reviewed. Different aspects will be highlighted as a combination of AMPs with antibiotics or organometallic compounds aiming to increase antibacterial activity or target selectivity, conjugation with photosensitizers for improving photodynamic therapy (PDT) or the attachment to particles, to name only a few. Owing to the enormous resonance of antimicrobial conjugates in the literature so far, this research topic seems to be very attractive to different scientific fields, like medicine, biology, biochemistry or chemistry.

## 1. Introduction

Growing antibiotic resistance has become a major health problem [1,2,3] and has encouraged many researchers to find alternative antibiotic classes. In this context, antimicrobial peptides (AMPs) have emerged as promising new agents to combat pathogens. AMPs are essential polypeptides in host defense and play an important role in the innate immune system [4,5,6,7]. They act against a diverse spectrum of organisms, such as Gram-positive and Gram-negative bacteria, as well as fungi, parasites and viruses [4,8,9]. AMPs exhibit a unique mode of action that is mainly related to their cationic amphipathic properties, making them capable of permeabilizing microbial membranes [4,5,8]. Accordingly, AMPs have come more and more into focus of research, although with the first clinical use of AMPs, the development of AMP-resistant strains is inevitable and intensively investigated [10,11].

### 1.1. Antimicrobial Peptides (AMPs): Classification

Antimicrobial peptides are highly diverse molecules, which can be divided into four groups based on structural characteristics: α-helical, β-sheet, extended and loop peptides [4,6,8]. Most AMPs share an amphipathic character with a net positive charge and a high content (around 50%) of hydrophobic residues. Magainin, LL-37 and cecropin [12,13] are examples of such AMPs that belong to the group of amphipathic α-helical peptides. The structure of β-sheet- or β-strand-like AMPs, including the defensin family and protegrin, is characterized by the presence of two or more disulfide bridges that stabilize their conformation [6]. Extended AMPs often contain a high percentage of proline, tryptophan, arginine and histidine residues in their primary sequence. In most cases, these peptides form only irregular secondary structures. Well-known representatives are indolicidin and bactenecins [14,15]. The smallest group of AMPs is the highly stable peptides exhibiting a hairpin-like loop structure that is interconnected by at least one disulfide bridge [16]. Examples include dodecapeptide [17] and tachyplesins [18].

During the last few years, many efforts have been made in the identification and development of synthetic AMPs [7,19]. The rationale is to shorten the size and thereby to optimize metabolic stability, bioavailability and issues regarding safety and immunogenicity. In addition, shorter sequences would dramatically decrease production costs. For instance, a detailed study about the critical length of cationic AMPs still exhibiting antimicrobial activity had been reported by Strom *et al.* some years ago [20]. Increased levels of activity might be also possible after the introduction of unnatural amino acids and replacement or modification of the peptide bond within the AMP sequence [21]. This review will also include some AMP conjugates composed of such designed AMPs.

### 1.2. AMPs: Mechanism of Action

Owing to their cationic nature, AMPs are attracted to the negative charges at the outer microbial membrane, supporting highly-selective interaction. Bacterial surfaces are characterized by their negative environment that is caused by the presence of negative compounds, such as lipoteichoic acid and lipopolysaccharides [22,23]. Compared to this, mammalian surfaces differ in their composition, containing zwitterionic phospholipids, sphingomyelin and cholesterol, which allows AMPs to selectively target microbial membranes [24]. However, the toxicity of AMPs against eukaryotic cells is an important issue and has to be investigated to realize future clinical applications [25,26,27]. For instance, the modulation of the physicochemical parameters, like hydrophobicity, net charge and helicity, might be a promising strategy [28] and has been investigated in several recent studies [29,30,31,32].

Usually, AMPs adopt well-defined secondary structures when they come into contact with the membranes of pathogens. After this initial step of binding by electrostatic forces, membrane permeabilization takes place, where bacterial membranes finally get disrupted [33]. This induces several processes, e.g., the breakdown of the membrane potential and the leakage of intracellular components, leading to subsequent cell death. The underlying membrane-disrupting mechanisms are highly complex and will be only briefly introduced. In particular, many different models have been suggested, including the toroidal-pore model, the barrel-stave model and the carpet-like model [34]. In the toroidal-pore model, the peptides aggregate on the membrane surface and induce the membrane to bend continuously through the pore, leading to a pore built up by the inserted peptides, as well as by the lipid head groups [6,35]. It is emphasized that the peptides, although being already inserted in the membrane, are continuously in association with phospholipid head groups [36]. In contrast, within the barrel-stave model, the attached peptides aggregate at the outer membrane first and, as a result, insert into the cell membrane [37,38]. Hereby, the hydrophilic peptide regions form the interior region of the core, and the hydrophobic part is oriented to the lipids of the cell membrane [36,39]. Lastly, the carpet-like model is characterized by the absence of such a pore formation. Peptides accumulate parallel to the bacterial membrane, covering the whole membrane surface in a carpet-like manner [40,41]. In the next step, the surface-attached peptides induce permeabilization, and the membrane is disrupted in a detergent-like manner, finally leading to the particular formation of micelles [42,43,44].

Although for most AMPs, membrane permeabilization processes are described, they can also act by using other mechanisms. After entering pathogenic cells, these AMPs reach intracellular targets, leading to the inhibition of cell-wall synthesis [45], inactivation of relevant enzymes [46,47,48] or affect DNA, RNA and protein synthesis [49,50,51,52].

## 2. Modified AMPs and AMP Conjugates

AMPs as a novel class of antimicrobial therapeutics give hope to confining the rise of antibiotic resistances and to acting as promising novel weapons against microbial pathogens. Moreover, since they are able to interact in some cases with human cell membranes, as well, AMPs can be used as delivery vectors for several bioactive compounds [53]. However, despite all of the benefits that have emerged with the development of AMPs, researchers aim to face several shortcomings and to enhance their antibacterial activity and mode of action by various modifications, leading to functionalized AMPs and AMP conjugates. The potential of such new AMP compositions, which include AMP mimetics, hybrid AMPs, AMP congeners, stabilized AMPs, immobilized AMPs and AMP conjugates, was recently summarized by Brogden *et al.* [54].

In fact, AMPs can be functionalized to generate peptide conjugates, in which various substances are attached by different coupling strategies. Very common is to fuse the modification at the α amino group of the N-terminal end with a carboxyl group to yield an amide bond. This can be done in many cases when the peptide is still attached to the solid support employing standard reagents that are used in solid phase peptide synthesis (SPPS). However, when the conjugate is generated in solution with the free peptide, chemoselective coupling methods have to be used, including activation by amino reactive *N*-hydroxysuccinimides (NHS), or thiol reactive maleimide groups, as well as copper(I)-catalyzed alkyne-azide cycloaddition (CuAAC), and many others. Within this work, we try to review recent advances in the generation and application of such AMP conjugates together with their interesting novel and modulated activities (Table 1).

### 2.1. Antibiotics Coupled to AMPs and AMP-Like Sequences

One strategy of AMP modulation is the covalent coupling of antibiotics to induce synergistic antibacterial effects. Such a combination therapy is supposed to increase antibacterial activity and to decrease administration dose, thus lowering the risk for adverse side effects. For example, vancomycin was coupled via CuAAC reaction to several peptide variants based on the magainin 2 sequence [76]. The authors tested the interaction with model membranes, bacterial growth inhibition and activity against eukaryotic membranes. Interestingly, an increase of antimicrobial activity of vancomycin-magainin conjugates was only visible against vancomycin-resistant *Enterococci*, where they determined minimal inhibitory concentration (MIC) values in the range or even better as for vancomycin alone. Besides, their results allowed concluding that the length of the peptide sequence had an influence on the mode of action with the bacterial membrane. Shorter peptides below 20 amino acids seemed to interplay via a carpet-like mechanism, while longer peptides preferably interacted by forming transmembrane pores. This observation was in agreement with former studies [42]. However, although the strategy seemed very promising since the conjugates showed no dramatic hemolytic effect, additive effects of both compounds in combination have not been tested yet.

Ghaffar *et al.* conjugated the broad spectrum antibiotic levofloxacin with the highly hydrophobic antimicrobial peptide indolicidin [65,91]. Coupling between both components was achieved via amide bond formation or a labile ester linkage to investigate whether a prodrug-type linkage would have any impact on activity. Although activity was still present, it could not be increased compared to the substances alone. Moreover, the activity was not dependent on the chosen linkage. Notably, the authors studied also the coupling to a cell-penetrating peptide (Tat_48-59_), but also in this case, no enhanced activity could be detected. Finally, the physical mixture of levofloxacin and indolicidin showed slightly improved antibacterial activity in comparison to levofloxacin and indolicidin alone [65], proving that covalent linkage had diminished the activity of levofloxacin.

Just recently, Chen *et al.* reported about a ubiquicidin (UBI) variant coupled to the typical antibiotic chloramphenicol (CAP) [86]. UBI is a cationic peptide that showed antibacterial activity towards *Staphylococcus aureus* [92]. UBI_29-41_ was used as a bacterial targeting sequence and demonstrated selective targeting capability against several bacteria (*Escherichia coli*, *S. aureus*, *P. aeruginosa*), as well as in bacteria-infected mouse models. Conjugation of CAP to the N-terminal of UBI_29-41_ was realized through the linker glutaraldehyde. The CAP-UBI_29-41_ conjugate showed enhanced activity in *E. coli* and *S. aureus*. Moreover, the toxicity against human cells was decreased compared to CAP alone. Most importantly, efficient targeting and bacterial killing was observed when analyzing the *in vivo* mouse model.

In several studies, it has been demonstrated that short peptide motifs can have a positive effect on the antibacterial activity of antibiotic drugs. For instance, Bera *et al.* developed a lysine-modified neomycin B variant to produce novel polycationic lysine mimetics that should increase antibacterial activity and simultaneously mediate higher binding affinity to RNA [90]. Neomycin B was chemically manipulated by taking advantage of the presence of a single OH-group at the C5′′-position. Thus, coupling of a short Tryp-Tryp-Lys-motif was possible. Recent studies, where neomycin was modified at this C5′′-position, have already proven that conjugates with interesting new antimicrobial activities can be obtained by this strategy [93,94,95]. Additionally, the neomycin B conjugates designed by Bera *et al.* demonstrated improved activity against resistant bacteria, such as methicillin-resistant *S. epidermidis* (MRSE) and gentamicin-resistant *E. coli*. Furthermore, it was hypothesized that attachment of amphiphilic amino acids might change the mode of action of neomycin B [90].

In another context, neomycin was modified with small dehydropeptides to yield amphiphilic nanostructures with a hydrophobic core and a hydrophilic surface [88]. The aim of the study was to design multifunctional amphiphilic conjugates capable of interacting with pDNA and the cell membrane, respectively. Besides using these conjugates as pDNA delivery vehicles, an application as novel antimicrobial agents seemed feasible. It was emphasized that the hydrophobic inner core and cationic outer surface of peptide-neomycin nanostructures would result in better electrostatic interaction with the bacterial membranes and that this might lead to an increased antimicrobial activity, as well. In this case, the activated peptide chain was introduced at the amino groups of neomycin. Indeed, characterization of the novel amphipathic peptide-neomycin conjugates proved that the hydrophilic outer surface of the nanostructures interacts specifically with bacterial membranes. With MIC values in the range of 8–9 μg/mL against Gram-positive bacteria, the novel peptide-neomycin nanostructures are highly versatile multifunctional carriers [88].

Taken together, these investigations exemplify that beside the positive effects, covalent coupling of antibiotics might diminish antibacterial activity. On the other hand, whenever the physical mixture of antibiotic and AMP was tested, an enhanced biological effect was determined. Accordingly, combination therapy itself is still very attractive, but the generation and activity of covalent conjugates has to be carefully explored in every case. For this, possible coupling sites within the structures of the investigated antibiotics should be identified to lower the risk of decreasing antibiotic efficiency after attachment to an AMP. Furthermore, novel ligation strategies should be tested, including also self-immolative linkers that liberate their cargo at the active site after a certain stimulus.

### 2.2. Improving AMP Activity by Lipidation

One other possibility of enhancing the activity of AMPs is to improve their interaction with the bacterial surface/membrane. Chu-Kung *et al.* reported about fatty acid-modified AMPs that were tested in terms of antibacterial activity and interaction with model membranes [56]. Fatty acids of different lengths (C_12_-C_20_) were introduced at the N-terminal end of AKK- and LKK-motif AMPs. The results indicated that the length of the fatty acid that can be introduced is limited, since too long acyl chains increased aggregation and self-assembly of the conjugates. However, conjugates, including C_14_-C_18_ long fatty acids, dramatically improved MIC values for several bacterial strains tested. It was concluded that linkage to fatty acids can promote the ability to form secondary structures when in contact with bacterial membranes [56,96].

The arginine/tryptophan containing peptide RWRWRW, named also MP196 [20], was modified with lipids of different lengths either at the N- or C-terminal end of the peptide [80]. Thus, an additional lysine residue was introduced N- or C-terminally, respectively, and the acyl chain was coupled to the ε-amino group of this lysine. Using this approach, assessment of both the length and attachment side of the lipid chain regarding bacterial and hemolytic activity was possible. Among all conjugates tested, a lipidated variant with a C_8_-acyl chain turned out to be the most effective. Interestingly, whereas the coupling site had no significant influence on the bacterial activity, hemolytic effects were strongly influenced. C-terminal lipidation was not that harmful, probably due to the fact that N-terminal lipidation combined with the positive charge of the N-terminus increased faster and more efficient hemolysis. In further studies, these C_8_-conjugates were investigated in more detail regarding their mode of action [97]. Particularly, the authors highlight in depth proteome profiling studies of the influence of the MP196 conjugates on the biological response of *B. subtilis*. Thereby, they could demonstrate for the first time that lipidation of MP196 does not change the mode of action considerably.

Recently, Arnusch *et al.* reported about the lipophilic modification of AMPs with the aim to narrow their mode of action and to direct their activity against fungi [89]. Lipopeptides form a class of peptides with a strong membrane affinity and, thus, broad activity spectrum. In this work, mimics of lipopeptides were generated by N-terminal coupling of different lipophilic groups, e.g., cholesterol, biotin or vitamin E, to short AMP sequences. Especially, vitamin E and cholesterol bioconjugates showed high and selective activities against the fungal strains tested, including *Aspergillus*, *Candida* and two more other species [89]. Then, cyclodextrin (CD) and amphotericin B (AmB) were tested for synergistic effects, whereby the addition of AmB led to a fungal inhibition concentration of 0.37 μM in *C. albicans*. However, the addition of CD did modulate the selectivity, particularly for the vitamin E-peptide conjugate. Summarizing, biologically-active lipopeptides with high membrane selectivity could be designed by this approach.

### 2.3. Photosensitizer-AMP Conjugates

Photodynamic therapy (PDT) uses a photosensitizer (PS) that, after exposure to a particular type of light, produces reactive oxygen species (ROS) that damage proteins, nucleic acids and lipids [98,99] and lead to cell death. PDT is particularly effective at killing resistant bacteria strains [98,100,101], whereas Gram-positive bacteria are more affected than Gram-negative bacteria, owing to their additional outer membrane [102,103,104]. Based on these observations, a combination of photosensitizers with AMPs was investigated by several groups to study additive biological effects. Johnson *et al.* tested whether amphiphilic AMPs conjugated to a PS bind and kill Gram-positive, as well as primary Gram-negative bacteria [66]. To reach this goal, the PS eosin Y was coupled as 5,6-carboxy eosin Y to the N-terminal of the AMP KLA (KLAKLAK)_2_. Both compounds showed no antibacterial effect when tested alone; however, in the presence of 1 μM of the conjugate, 99% of the tested bacteria were killed. Furthermore, conjugation of (KLAKLAK)_2_ to eosin Y enhances binding to bacteria 10-fold. Of note is that only 10% hemolysis was obtained, demonstrating that the photolytic activity is more pronounced towards bacteria than to eukaryotic cells. Altogether, these results highlighted eosin-(KLAKLAK)_2_ as a promising new lead compound for bacterial treatment. In further experiments, the additive effects of eosin and the AMP were elucidated in more detail [67]. It was found out that the PS-peptide was localized at Gram-negative and Gram-positive bacterial membranes, respectively, and upon irradiation, the eosin-(KLAKLAK)_2_ conjugate was capable of destroying Gram-positive, as well as Gram-negative bacterial membrane components. Based on these results, it was reasoned that the (KLAKLAK)_2_ part acts as a targeting agent to bacterial membranes and disrupts the membrane by oxidation of lipids or that the lytic activity of oxidized lipids is amplified by the AMP component [67].

Porphyrins are another group of photosensitizers that were coupled to AMPs. It has been documented that neutral PS kill Gram-positive bacteria, but do not kill Gram-negative bacteria [105,106,107]. Doselli *et al.* tested in 2010 a porphyrin-apidaecin conjugate [55]. Also in this study, attachment of the PS took place at the N-terminus of the AMP. Notably, when performing toxicity studies in the dark, the conjugate completely lost its antibacterial activity. Contrarily, the compounds alone were still active. This observation probably suggests that the size of porphyrin prevented the uptake of the conjugate inside the bacteria cells. Interestingly, the light-activated conjugate was able to kill bacteria and exhibited high antibacterial activity, especially against *E. coli* and *S. aureus* [55].

Liu and co-workers reported effective photodynamic therapy by using a conjugate consisting of protoporphyrin (PpIX) coupled to two units of a lipopolysaccharide binding AMP to selectively adhere to the surface of Gram-negative bacteria. In this study, the authors activated PpIX with a maleimide group for selective coupling with the sulfhydryl group of a cysteine of the AMP, named YI13WF. Subsequently, they used this conjugate for specific imaging and photodynamic inactivation of Gram-negative bacteria [87], and their results proved the successful delivery of the photosensitizer to the bacterial surface. In addition, four- to eight-fold lower MIC values of the PpIX-peptide conjugate compared to the peptide and PpIX alone were obtained. Still, PpIX-peptide conjugates demonstrated selective targeting against bacterial strains only, since no activity against mammalian cells was detected [87].

All PS-AMP conjugates discussed herein exhibited increased activities compared to the parent compounds alone. AMPs were mostly used to provoke selective binding to the pathogens, while by the attached PS, the production of ROS was induced. All in all, the combination of PS with AMPs seems to be a highly promising approach to improve antibacterial activity and to widen the overall activity spectrum of such compounds.

### 2.4. Decoration of Particles and Polymers with AMPs

Nano-/micro-particles can be produced out of a large variety of materials, resulting in different shapes, sizes and surfaces. During the last few years, one main field of research was their development and application as drug delivery vehicles [108]. Furthermore, attachment to particles can be used to avoid any dissemination of AMPs to the environment. For instance, paramagnetic silica microparticles were surface-modified by magainin-I [75]. The particles were functionalized by a hydrophilic polymer brush, and the AMP was grafted via a heterofunctional linker to the hydroxyl groups on the surface. In this way, multifunctional particles exhibiting antibacterial and magnetic properties could be obtained. Such conjugates might be interesting for the disinfection of aqueous solutions, but also for *in vivo* applications due to the possibility of directing them by magnetic fields to a localized antibacterial action.

Coating of surfaces with AMPs aiming to reduce or prevent the formation of biofilms infection is also a growing field of interest, particularly in the biomedical field [38]. Usually, hard surfaces made of glass or titanium are decorated with AMPs in an often multi-step synthesis, starting with surface functionalization by appropriate linkers, followed by conjugation of the AMPs. Many of such routes have been described; however, the details are out of the scope of this review. Despite the attachment of the AMPs directly onto the surface, Rai *et al.* recently reported about AMP-decorated gold (Au) nanoparticles that were coated on glass slides [59]. There, the Au-particles were functionalized with a maleimide linker to which the thiol group of a cecropin-melittin AMP was attached. The AMP-Au particles were then coated on glass surfaces, and bacterial activity against *E. coli* and *S. aureus* was tested. The authors report high activity against both strains tested, which was presumably due to a higher coating density compared to other studies reported so far. Importantly, the antibacterial activity of the surface immobilized AMP was more effective in long-term exposure compared to the free AMP. Additionally, no toxicity against human cells was detected.

To minimize or reduce microbial contamination on polymer surfaces, AMPs can be directly introduced onto the polymer scaffold by covalent coupling. Recently, Kim *et al.* reported about AMPs immobilized on PEG-resin [70]. They used an antibacterial β-sheet peptide and an α-helical peptide that were both immobilized on PEG-PS resin. Both conjugates were tested against bacterial strains, but only the β-sheet peptide containing resin exhibited antibacterial activity. Again, the activity could be increased by the addition of common antibiotics like vancomycin or tetracycline, respectively, proving their approach as a powerful strategy to enhance antibacterial activity. Since such surface immobilization of AMPs might be critical due to the effect on the secondary structure during the immobilization process, Gao *et al.* investigated the membrane interaction of surface-tethered AMP IDR-1010cys [64]. IDR-1010cys, short-chain antimicrobial and immunomodulatory peptide innate defense regulator (IDR) 1010, was immobilized via maleimide linkage to functionalized quartz slides. Several physical parameters were determined; importantly, the influence of immobilization on the secondary structure, as well as the ability to interact with model membranes was elucidated. Compared to the free peptide in solution, their results allowed concluding that biological activity is extremely dependent on the surface density of the peptides. In addition, the chosen linkage between the AMP and the surface can have a profound impact and should be selected carefully. This observation holds true also for other studies where AMP immobilization was investigated [109,110].

Knowledge about the mechanism of action of AMPs is indispensable for future development. Especially, *in vitro* measurements do not necessarily correlate with the *in vivo* activity of antimicrobial peptides. Recently, a novel fluorescent live bacteria lysis assay was developed by Leptihn *et al.* [83]. In this study, the process of antibacterial action was monitored on the single-molecule level. Sushi I, an α-helical cationic AMP that targets Gram-negative bacteria, was coupled at the N-terminal to biotin and coated on streptavidin-modified quantum dots. Analyzing those quantum dot-Sushi I conjugates, it could be shown that they bind to the bacterial surface and migrate over the membrane surface. Increased concentrations led to decreased movement of the peptide, indicating a multimeric association of Sushi I and the formation of membrane-active peptide complexes. Additionally, membrane lysis was proven by single particle tracking and bacterial cell lysis assays. Furthermore, the authors generated nanogold-Sushi I conjugates that were inspected using transmission electron microscopy (TEM). Their observations indicated that sushi I binds to the outer and inner membrane of *E. coli* bacteria, leading finally to the leakage of cytosolic content [83]. Although such studies allow the dissemination of AMP molecular mechanisms, it has to be proven accurately how the results can be transferred to other structurally-similar AMPs.

Despite these more specific examples, recent efforts on AMP-nanoparticle combinations have been made in the context of clinical development of AMPs with the aim to lower cytotoxicity, increase proteolytic stability and improve antimicrobial activity. Thus, nanoscale particles have been used to include AMPs in novel formulations [111].

### 2.5. Conjugates out of AMPs and Organometallic Complexes

During the last few years, the introduction of organometallic groups in biomolecules, like peptides, proteins or nucleic acids, has come more and more into the focus of research. The metal center can cause novel functionalities and reactivities to the new compounds, and among them are also substances showing promising effects against multidrug-resistant bacteria [112]. For example, ferrocene (Fc) is one of these compounds for which it is known that it can enhance the pharmacodynamic profiles of bioactive molecules [113]. Recently, Fc was combined with the AMP MP66 [114], a small peptide fragment that was derived from the protein lactotransferrin [79]. Ferrocene was coupled via amide bond formation to the N-terminal of MP66. It was shown that treatment of *C. glutamicum* with the ferrocene-MP66 conjugate causes cellular stress. Furthermore, a specific cell response related to the Fc moiety was detected that was expressed in an upregulation of cell-wall synthesis proteins and downregulation of permeases and porins. Additionally, the lipid composition of the cell was drastically altered. The herein reported approach gave for the first time access to metal-conjugated peptide antibiotics with membrane-targeting properties [79].

Recently, Pal *et al.* reported about a multifunctional non-covalent complex including antimicrobial, as well as wound healing activity. It consisted of a bi-valent silver polydiguanide complex including histatin-1 [62], an AMP that was identified to be involved in re-epithelialization processes [115]. The synthesis of the metal complex-containing peptide conjugate was achieved by complexation between a silver chlorhexidine complex and the peptide. The combination of both compounds demonstrated promising MIC values (around 1 mg/L) against a wide spectrum of bacteria and, furthermore, was identified as a wound healing-promoting agent. In the future, such combinations might be useful for the treatment of infected wounds [62].

In recent years, biosensors played an important role in pathogenic bacteria detection. Yongxin *et al.* used a ferrocene-peptide conjugate to develop a novel metal-containing biosensor [74]. Owing to its favorable electrochemical properties, Fc and derivatives have been often used in electrochemical systems. The surface of the electrode was functionalized with an NHS linker to couple the ferrocene derivative that contained two carboxyl groups, via an amide bond. Magainin I, for which an enhanced selectivity of bacterial recognition has been already reported [116], was coupled to the remaining carboxyl group of Fc. The resultant ferrocene-magainin conjugate was then used to detect *E. coli* cells. Notably, the ferrocene-magainin biosensor induced a 10-fold increased detection limit of *E. coli* compared to label-free biosensors, making this approach promising for future bacterial detection.

In another study, selective labeling of tryptophan residues by organometallic ruthenium complexes was used to elucidate the biodistribution of the AMP melittin in mice [78]. Melittin is a component of bee venom and has attracted much attention owing to its antibacterial and anticancer activity [117]. Complexation of melittin with the commercially-available ruthenium complex [(C_5_H_5_)Ru(naphthalene)]^+^ in aqueous solution and in air gave the organometallic derivative of melittin, in which ruthenium was coordinated via a tryptophan residue of the peptide. The ruthenium-containing label showed high chemical stability and had no influence on the secondary structure of the peptide. However, *in vivo* tracking of the ruthenium-labeled AMP was possible, and the labeling changed the intramolecular interactions of the AMP in that it had reduced hemolysis activity [78].

### 2.6. AMPs and AMP Containing Conjugates as Anti-Cancer Drugs

Recently, it has been recognized that cationic antimicrobial peptides can serve as novel drugs with cancer-selective activity. Cancer cells are characterized by a higher content of anionic phospholipids, such as phosphatidylserine, at the outer leaflet, making them attractive targets for the mainly positively-charged AMPs. After binding, they can generate physical holes causing leakage of the cell content and, finally, cell death [118]. To further improve their selectivity and to reduce side effects to healthy cells, Rivero-Mueller *et al.* generated a peptide conjugate composed of an AMP and a cancer-targeting peptide [60]. Such hybrid peptides including a targeting domain and a cell-killing domain are usually obtained by direct chemical peptide synthesis or as fusion protein expressed in a recombinant system. In this study, hecate, an analogue to the bee venom main component melittin, was chemically fused with the 81–95 amino acid fragment of the chorionic gonadotropin-β (CGβ) subunit. This CGβ subunit is known to target cells expressing the luteinizing hormone receptor (LHR), even at very low doses or when LHR is expressed at low levels [60]. Testing the hecate-CGβ conjugate in mouse xenografts and transgenic mouse models proved its efficiency in destroying LHR-expressing cancer cells. The mechanism of action was the destruction of the cell membrane after binding to the LHR-expressing cells leading to cell necrosis with only minimal side effects. In a similar study, the reactivity of magainin II was directed against cancer cells by fusion to bombesin that targets selectively receptors overexpressed on various kinds of cancer cells [77]. The fusion peptide was investigated *in vitro* and *in vivo* and demonstrated promising anticancer activity.

Furthermore, for the peptide buforin IIb cancer-targeting specificity was enhanced, in this case by fusion to a matrix metalloproteinase (MMP)-selective cleavage site. MMPs are zinc-dependent endopeptidases, and many cancer types have shown increased expression levels of MMPs. Furthermore, buforin was masked by an anionic peptide to decrease side effects deriving from the cationic charges of buforin [58]. Indeed, the conjugate showed improved effects compared to buforin alone when different cancer cells were treated.

Recently, hecate was modified with gallic acid, and the activity against cancer and non-cancer cells was tested [61]. Gallic acid was introduced at the N-terminal by standard solid phase peptide chemistry. Interestingly, although the α helical content of the secondary structure was reduced after introducing gallic acid to hecate, activity against erythrocytes was increased. The authors discussed this with the increased hydrophobicity and hydrophobic moment that was already shown to enhance the lysis of erythrocytes [119].

In this context, Zhong *et al.* reported about a polyvalent lytic peptide-polymer conjugate that was designed to overcome the problem of multidrug resistance. The lytic hexapeptide KWKWKW, or KW_3_, was coupled to dextran in several copies via CuAAC chemistry. The resultant KW_3_-dextran conjugates showed 500-fold increased anticancer potency compared to the hexapeptide alone and even promising effects in multidrug-resistant cancer cells. Again, no hemolytic activity was observed [71]. It was reasoned that their high potency against cancer cells was caused by the polyvalent structure that increased the local concentration of the membrane-active peptide.

One other peptide for which first antimicrobial activity and later on also anticancer activity have been reported is the amphipathic peptide KLA ([KLAKLAK]_2_) that destabilizes the mitochondrial membrane potential and triggers the apoptotic cell death program [120]. Meanwhile, several examples of hybrid peptides have been described where KLA as the proapoptotic domain was coupled to other carrier peptides, like cell-penetrating or tumor-homing peptides [81,121,122]. Moreover, in 2003, Jiang *et al.* conjugated the protein transduction domain PTD-5 to KLA with the aim to produce a pro-apoptotic peptide (named DP1), which should selectively disrupt both mitochondrial and bacterial membranes [68]. PTD-5 has been reported to be very efficient in internalization into cells [123]. Western blots of mitochondrial and cytosolic fractions indicated cytochrome c (cyt c) release from mitochondria after treatment with DP1. Additionally, the release led to enhanced H_2_O_2_ production and to selective oxidation of phosphatidylserine in the inner leaflet of the plasma membrane during apoptosis. Collectively, the cyt c release plays an important role in selective catalysis of phosphatidylserine oxidation. Finally, DP1 induced phosphatidylserine externalization and enhanced phagocytosis of treated cells by macrophages [68]. In another study, KLA was conjugated to octaarginine or to the hydrophobic PFVYLI peptide, in both cases to improve the delivery of KLA inside cells. A cellular localization assay demonstrated that PFVYLI is internalized in cells in a temperature-dependent way by endocytosis. When using this peptide as a transporter for KLA, a significant influence on cell viability was detected. Although the uptake mechanism remained undetermined, it seemed to be different than for the octaarginine compound [69].

Soler *et al.* investigated the ability of several AMP variants to act as delivery vehicles for the cytostatic drug chlorambucil [57]. A set of peptides selected from a library of cecropin-melittin hybrids (CECMEL11), previously designed to be used in plant protection, was screened for their cytotoxicity against diverse cancer cell lines. The best peptide exhibiting no effects on cell viability was BP16, which was then tested for its ability to transport chlorambucil inside cells. The drug was covalently coupled by amide bond formation at the N-terminal of BP16, and its activity against several cancer cell lines was elucidated. It could be shown that attachment to BP16 dramatically increased the cytotoxicity of this drug between six- and nine-fold. Additionally, when in conjugation with the homing peptide CREKA, BP16 was able to improve the cytotoxic activity of chlorambucil from two- to 4.5-fold, demonstrating again the positive affect of such a coupling.

In our group, we developed a branched peptide variant of the peptide sC18, a short C-terminal fragment of the 18-kD antimicrobial protein CAP18 [124,125]. This branched dimer of sC18, namely (sC18)_2_, was used as a drug delivery vector for chlorambucil or KLA, respectively [81]. In both cases, we introduced a cathepsin B cleavage site between the cargo and the peptide carrier. Cathepsin B is a protease that is overexpressed in many tumor cells and, thus, allows one to promote selective cargo release at the target site [126,127]. We could demonstrate that (sC18)_2_ was an efficient transporter and that it exhibited certain selectivity against cancer cells, which we attributed to its increased content of hydrophilic residues compared to the parent peptide sC18. Thus, improved interaction with the anionic membranes of cancer cells is possible [81].

### 2.7. Other Examples of Modulated AMPs

Using CuAAC reaction coumarin, a benzopyrone with antimicrobial, anti-inflammatory and anticancer properties was conjugated to UBI [128]. The combination of both parts displayed antimicrobial activity within a concentration range of 0.04–0.18 mM. Furthermore, it was not toxic to human cells above 0.21 mM [84]. The same peptide was used in another context to develop a hybrid tracer useful for SPECT and optical imaging of bacterial infections [85]. Welling *et al.* modified UBI both with a Cy5 dye and a DTPA chelator useful to complex the radioisotope ^111^In. The hybrid label was introduced when the peptide still resided in the solid phase, and the resultant hybrid conjugate was tested *in vitro* and *in vivo*. Notably, the used peptide had superior properties for binding multiple bacteria and, therefore, serves as an excellent imaging and detection agent.

In a previous work, we investigated the influence of imidazolium cations on the activity of AMPs [72,73]. Based on observations that imidazolium cations, as part of so-called ionic liquids [73,129], exhibit antimicrobial activity, these compounds were covalently and non-covalently added to AMPs. AMPs used for coupling were sC18 [125] and the LL-37 peptide, which also belongs to the group of cathelicidins and is a two-amino acid truncated form of FALL-39 [13,130]. The imidazolium salts were coupled to the α- and ε-amino groups of lysine residues that were N-terminally elongated to the peptide. The resultant compounds exhibited high antimicrobial activity (around 1 µM) against different Gram-positive and Gram-negative bacterial strains and even against multidrug-resistant strains, like MRSA and VRE. Notably, non-covalent mixtures were not as efficient as the covalently-bridged constructs. Furthermore, one short AMP-IL-conjugate, IL-KKA, was identified that also showed promising selectivity towards bacterial membranes compared to eukaryotic ones [72,73].

Another modification to improve bacterial selectivity and activity was the coupling of sheep myeloid antimicrobial peptide (SMAP) 28 to affinity- and protein G-purified rabbit immunoglobulin G (IgG) antibodies specific to the outer surface of *Porphyromonas gingivalis* strain 381 [82]. The linkage between both moieties was performed by using a chemoselective cross-linking reaction between maleimide that was attached to SMAP 28 and the sulfhydryl groups of the antibody. Franzmann and co-workers could demonstrate that the selectivity of the antibody-AMP conjugate was concentration dependent and that the best results were obtained when concentrations of 20 µg protein/mL were applied. Thus, they developed a highly potent and selective conjugate against *P. gingivalis*.

In another study, Tati *et al.* investigated spermidine-AMP conjugates to function as inhibitors against oral *Candida* strains. Spermidine was linked to an active fragment of histatin 5 that belongs to the histatins, a family of histidine-rich cationic peptides secreted by human parotid and submandibular-sublingual salivary glands with selective antifungal activity and little or no bactericidal activity [63]. Two different variants were generated, one having the spermidine unit at the N-terminal, the other at the C-terminal end. The authors reported that topical treatment of oral candidiasis with the His-spermidine conjugate was highly effective. In particular, doses well below those of the control fluconazole could be applied. Interestingly, placement of spermidine had a profound impact on activity, since the conjugate bearing spermidine at the C-terminal end was significantly more active.

Other applications of AMPs include their use as escaping peptides for endocytosis-mediated drug delivery. In these cases AMPs are often fused to CPPs, and functionalized with the respective cargo. For instance, Salomone *et al.* combined the cell-penetrating peptide TAT_11_ with the AMP hybrid CM18 (derived from cecropin A and melittin) and investigated its drug delivery properties. Improved endosomal drug release was observed by using this strategy [131]. Moreover, using a conjugate composed of the designed AMP, C(LLKK)3C, fused to the TAT peptide and stearic acid, delivery of nucleic acids was determined. Promising transfection efficiencies were observed, leading to the conclusion that the conjugate was efficiently released out of the endosomes by its capacity to lyse the endosomal membranes [132].

## 3. Concluding Remarks

Overuse of common antibiotic drugs in hospitals, but also in industrial farming, has provoked the emergence of multidrug-resistant organisms. To efficiently combat these pathogens, finding novel classes of antibiotics has become a major global concern and has been pushed forward over the last few years. Within this field of research, antimicrobial peptides have become key players as promising new alternative therapeutics. Beside their application as natural sequences, many efforts were made in the generation of AMP conjugates to create compounds with novel activity spectra. Looking back on the last few years, many new groups of conjugates have been identified, and interesting new molecules have been designed, not only with the rationale to enhance the efficiency against bacterial pathogens, but also to use AMPs as, e.g., anticancer drugs. Regarding the forthcoming improvements in synthesis strategies to lower production costs, such AMP conjugates might be relevant future solutions with high potential.

## Figures and Tables

**Table 1:** Overview of AMP conjugates discussed in this study. AMPs are listed in alphabetical order.

Antimicrobial Peptide and Sequence	Conjugated Group	Coupling Method	Strains and Organisms Tested	Ref.
**Apidaecin Ib**	Porphyrin	Amide bond	*Escherichia coli* (ATCC 25922); *P. aeruginosa* (ATCC 25668); *MRSA* (ATCC BAA 44)	[55]
**GNNRPVYIPQPRPPHPRL**				
**AKK-motif/LKK-motif**	Fatty acids	Amide bond	*E. coli* (DH5α, ML-35); *S. epidermidis* (ATCC 12228)	[56]
**YGAA(KKAAKAA)_2_/YG(AKAKAAKA)_2_**				
**BP16**	Chlorambucil	Amide bond	3T3; MCF-7; CAPAN-1	[57]
**KKLFKKILKKL**				
**Buforin IIb**	Targeting peptide	Amide bond	FSF; HeLa; B16-F0; HT1080; U87MG	[58]
**RAGLQFPVG(RLLR)_3_**				
**Cecropin-melittin**	Au particles	Thioether	*S. aureus* (ATCC 6538); *E. coli* (ATCC 25922); *K. pneumoniae* (ATCC 10031); *P. aeruginosa* (ATCC 15442); multidrug-resistant *E. coli*; *S. haemolyticus* HUVECs; NDHF	[59]
**KWKLFKKIGAVLKVL**				
**Hecate FALALKALKKALKKLKKALKKAL**	Chorionic gonadotropin-β	Amide bond	Murine Leydig tumor BLT-1 cells	[60]
	Gallic acid	Amide bond	HeLa; HaCat	[61]
**Histatin-1**	[Ag(II)CHX]	Complex conjugate	*A. calcoaceticus (*ATCC 23055); *C. freundii* (ATCC 6750); *K. pneumonia* (ATCC 10031); *P. aeruginosa* (ATCC 27853); *E. faecalis (*ATCC 29212); *S. aureus* (ATCC 25923); *S. epidermis* (ATCC 12228); *P. bacterium acnes* (ATCC 6919) 3T3-L1 preadipocyte	[62]
**DSHEKRHHGYRRKFHEKHHSHREFPFYGDYGSNYLYDN**				
**Histatin-5**	Spermidine	Amide bond	*C. albicans* (CAF4-2); *C. glabrata* (931010, 90032, 90030)	[63]
**DSHAKRHHGYKRKFHEKHHSHRGY**				
**IDR-1010cys**	Quartz slides	Thioether	*P. aeruginosa*	[64]
**IRWRIRVWVRRIC**				
**Indolicin**	Levofloxacin	Amide bond/ester linkage	*E. coli* (ATCC 11775); *P. aeruginosa* (ATCC 10145); *S. aureus* (ATCC 25923, ATCC 9144); *B. subtilis* (ATCC 6051, ATCC 6633)	[65]
**ILPWKWPWWPWRR**				
**KLA, proapoptotic domain peptide (KLAKLAK)_2_**	Eosin Y	Amide bond	*S. aureus* (8325-4, ATCC 29213); *S. pyogenes* (12202); *P. aeruginosa* (PA01); *E. coli* (ATCC 25992; BL21 DE3)	[66,67]
	PTD-5	Amide bond	MCA-205 murine fibrosarcoma line; human head and neck tumor clinical isolates (22B and 4129)	[68]
	Octaarginine/PFVYLI	Amide bond	KG1a; HeLa	[69]
**KSL7/KSL8**	PEG-PS resin	Amide bond	*M. luteus* (ATCC 9341); *S. aureus* (ATCC 6538); *E. coli* (ATCC 25922); *P. aeruginosa* (ATCC 9027)	[70]
**FKVKFKVKVK/ LKVVFKVLFK**				
**KW_3_**	Dextran	CuAAC	MCF-7, PC-3, NIH/3T3, MES-SA, MES-SA/Dx5	[71]
**KWKWKW**				
**LL-37**	Imidazolium salt	Amide bond	*B. subtilis* (ATTC 6633); *E. coli* K12 (MG 1625); *M. phlei* (DSM 48214); *MRSA*; *VRE*	[72,73]
**LGDFFRSKEKIGKEFKRIVQRIKDFLRNLVPRTES**				
**Magainin I**	Ferrocene	Amide bond	*E. coli* K12; *S. epidermidis*; *B. subtilis*	[74]
GIGKFLHSAGKFGKAFVGEIMKS	Silica particles	Sulfide bond	*L. ivanovii*	[75]
**Magainin II**	Vancomycin	CuAAC	*MRSA* (15A761, 15A763); *VSE* (15A797); *VRE* (15A799); *M. catarrhalis* (58L028)	[76]
GIGKFLHSAKKFGKAFVGEIMNS	Bombesin	Amide bond	MCF-7; ZR-75-30; A375; M14; A875; DU145; HeLa; A549; Raji; NB4; Vero E6; Hek-293A, HSF; HUVECs; hPBMCs	[77]
**Melittin**	[(C_5_H_5_)Ru]^+^	Complex	SKOV_3_, MDA-MB-231, CBA mice	[78]
**GIGAVLKVLTTGLPALIS**				
**MP66**	Ferrocene	Amide bond	*C. glutamicum* (ATTC 13032)	[79]
**WRWRW**				
**MP196**	Lipids	Amide bond	*E. coli* (DSM 30083); *A. baumannii* (DSM 30007); *P. aeruginosa* (DSM 50071); *S. aureus* (DSM 20231, ATCC 43300); *B. subtilis* (168 DSM 402) MCF-7; HT29; Fibroblast (GM5657)	[66,80]
**RWRWRW**				
**Tat_48-59_**	Levofloxacin	Amide bond/ester linkage	*E. coli* (ATCC 11775); *P. aeruginosa* (ATCC 10145); *S. aureus* (ATCC 25923, ATCC 9144); *B. subtilis* (ATCC 6051, ATCC 6633)	[65]
**GRKKRRQRRRPQ**				
**sC18**	Imidazolium salt	Amide bond	*B. subtilis* (ATTC 6633); *E. coli* K12 (MG 1625); *M. phlei* (DSM 48214); *MRSA*; *VRE*	[72,73]
**GLRKRLRKFRNKIKEK**				
**(sC18)_2_**	Chlorambucil/KLA	Amide bond	HEK-293; MCF-7; HT-29	[81]
**GLRK(GLRKRLRKFRNKIKEK)RLRKFRNKIKEK**				
**SMAP28**	IgG	Thioether	*P. gingivalis* (381); *A. actinomycetemcomitans* (FDC-Y4); *P. micros*	[82]
**RGLRRLGRKIAHGVKKYGPTVLRIIRIA**				
**Sushi I**	Quantum dot/nanogold	Biotin/streptavidin	*E. coli* (ATCC 25922)	[83]
**GFKLKGMARISCLPNGQWSNFPPKCIRECAMVS**				
**Ubiquicidin_29-41_**	Coumarin	CuAAC	MDA-MB-435; female athymic nude mice	[84]
**TGRAKRRMQYNRR**	Hybrid label of Cy5 dye and DTPA chelator	Amide bond	*S. aureus* (ATCC 29213); *S. epidermidis* (ATCC 12228); *K. pneumoniae* (ATCC 43861); *E. coli* (ATCC 25922); *B. subtilis* (JH642) GE11-β3 Swiss mice	[85]
	Chloramphenicol	Amide bond	*S. aureus; E. coli; P. aeruginosa; B. subtilis;* L-02; HBL-100; HELF athymic nude mice; normal mice	[86]
**YI13WF**	Protoporphyrin IX	Thioether	*E. coli* DH5a (ATCC 53868); *S. enterica* (ATCC 14028); *E. coli* BL21 (Amp^r^*E. coli*); *K. pneumoniae* (ATCC 700603); Jurkat T cells	[87]
**YVLWKRKRKFCFI**				
**Other AMP like sequences**				
**Dehydropeptide**	Neomycin B	Amide bond	*E. coli* MG1655 (MTCC 1586); *P. aeruginosa* (MTCC 741); *S. typhimurium* (MTCC 98); *B. subtilis* (MTCC 121, MTCC 430); *S. aureus* (MTCC 740)	[88]
**Boc-FΔF-εAhx**				
**Lysine based tetrapeptides**	Biotin, vitamin E, cholesterol	Amide bond	42 different bacterial and fungal strains (especially *Aspergillus* and *Candida*)	[89]
**WWK-motif**	Neomycin B	Amide bond	*S. aureus* (ATCC 29213); *MRSA* (ATCC 33592); *S. epidermidis* (ATCC 14990); *MRSE* (CAN-ICU 61589); *S. pneumoniae* (ATCC 49619); *E. coli* (ATCC 25922; CAN-ICU 61714); *P. aeruginosa* (ATCC 27853; CAN-ICU 62308)	[90]

## Abbreviations

3T3	Mouse fibroblast cells
AmB	Amphotericin B
AMP	Antimicrobial peptide
B16-F0	Mouse skin melanoma cells
Bac	Bacitracin
BLT-1	Murine Leydig tumor cells
CAP	Chloramphenicol
CAPAN-1	Human pancreatic adenocarcinoma cells
CBA	Mouse kidney cells
CD	Cyclodextrin
CuAAC	Copper(1)-catalyzed alkyne-azide cycloaddition
Cyt c	Cytochrome c
DTPA	Diethylene triamine pentaacetic acid
DU-145	Human prostate carcinoma cells
DX5	Antibody that reacts with CD49b integrin
Fc	Ferrocene
FSF	Human facial skin fibroblast cells
GE11-β3	Mouse mammary epithelial cells
HT-1080	Human fibrosarcoma cells
IDR	Immunmodulatory peptide innate defense regulator
IgG	Immunglobuline
IL	Ionic liquid
KG1a	Human bone marrow macrophages
KLA	Proapoptotic domain peptide (KLAKLAK)_2_
LPS	Lipopolysaccharide
MBP	Maltose binding protein
MCA-205	Murine fibrosarcoma cells
MCF-7	Michigan Cancer Foundation-7, breast cancer cell line
MES-SA	Human uterine epithelial cells
MIC	Minimal inhibitory concentration
MMP	Matrix metallo-proteinase
MRSA	Methicillin-resistant *S. aureus*
MRSE	Methicilin-resistant *S. epidermidis*
NDHF	Normal human dermal fibroblast cells
NHS	*N*-hydroxysuccinimide
pDNA	Plasmid desoxyribonucleic acid
PDT	Photodynamic therapy
PEG-PS	Polyethylene glycol-polystyrene
PNA	Peptide nucleic acid
PpIX	Protoprophyrin IX
PS	Photosensitizer
RNA	Ribonucleic acid
ROS	Reactive oxygen species
SCOV	Human ovary adenocarcinoma cells
SDS PAGE	Sodium dodecyl sulfate polyacrylamide gel electrophoresis
SMAP	Sheep myeloid antimicrobial peptide
SPECT	Single-photon emission computer tomography
SPPS	Solid phase peptide synthesis
TEM	Transmission electron microscopy
U87 MG	Human glioblastoma cells
UBI	Ubiquicidin
VRE	Vancomycin-resistant *Enterococci*
ZR-75-30	Human breast ductual carcinoma cells
